# The state of the research for health environment in the ministries of health of the Economic Community of the West African States (ECOWAS)

**DOI:** 10.1186/1478-4505-11-35

**Published:** 2013-09-11

**Authors:** Issiaka Sombié, Jude Aidam, Blahima Konaté, Télesphore D Somé, Stanislas Sansan Kambou

**Affiliations:** 1 West African Health Organisation, 01 BP 153, Bobo-Dioulasso 01, Burkina Faso; 2 Centre MURAZ, 01 BP 390, Bobo-Dioulasso 01, Burkina Faso

**Keywords:** National research systems, Research environment, Research for health, Research governance, Research management, West Africa

## Abstract

**Background:**

An assessment of the state of the Research for Health (R4H) environment can provide relevant information about what aspects of national health research systems needs strengthening, so that research output can be relevant to meet national priorities for decision-making. There is limited information on the state of the R4H environment in the Economic Community of West African States (ECOWAS). This article describes the state of the R4H environment within the Ministries of Health of the ECOWAS member states and outlines of some possibilities to strengthen health research activities within the ECOWAS region.

**Methods:**

Information on the national-level R4H environment (governance and management; existence of a national policy; strategic and research priorities documents; ethics committees; research funds; coordination structures; monitoring and evaluation systems; networking and capacity building opportunities) was collected from the Ministries of Health research units in 14 ECOWAS countries using self-administered questionnaires. A workshop was held where country report presentations and group discussions were used to review and validate responses. Data from the discussions was transcribed using Nvivo, and strengths, weaknesses, opportunities and threats (SWOT) analysis of the functioning of the units was done using Robert Preziosi’s organisational diagnosis tool.

**Results:**

The findings indicate that as of January 2011, 50% of ECOWAS countries had established directorates for health research with defined terms of reference. The existing funding mechanisms were inadequate to support the research structures within and outside the MoHs, and for building the capacity of researchers. Networking and monitoring activities were weak and only 7% of the directors of research units were trained in research management. The majority (85.7%) of countries had broader national health policies, and 57% of the countries had some form of policy or strategic document for research development. Half of the countries had developed national research priorities.

**Conclusions:**

These results call for urgent action to improve the research environment in the Ministries of Health in the West African sub-region.

## Introduction

Research for health and socioeconomic development is considered an essential national investment and should be given all the necessary resources and attention to enable the determination of the causes and viable solutions to problems and inventions. The World Health Organization (WHO) has recognized the role of research in general and research for health (R4H) in particular in the fight against diseases and support for socioeconomic development [1], as stated in Article 2 of its 1946 Constitution: ‘boost and guide research in the area of health’ [2]. More recently, the Algiers Declaration and the Bamako Declaration with its implementation framework in the African region, called on all Ministers of Health to give prominence to research in their programmes [3-5].

**Figure 1:**
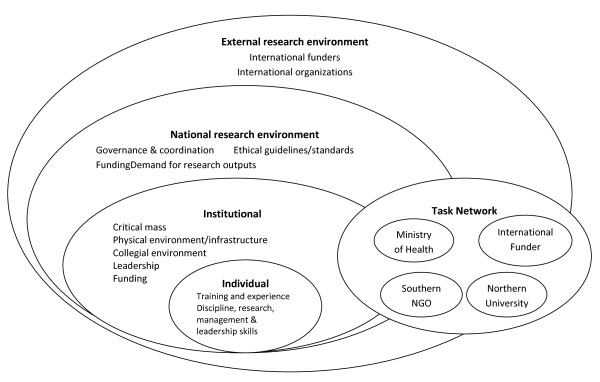
**Research capacity conceptual framework created by McIntyre [**[6]**].**

**Figure 2:**
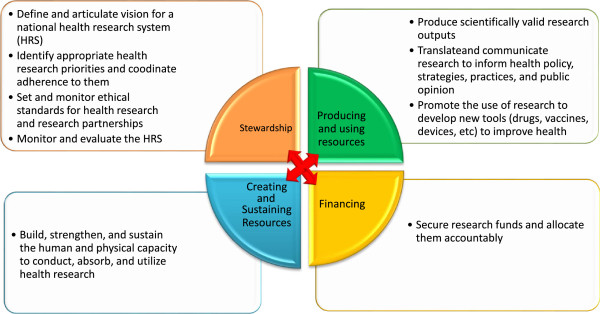
**A conceptual framework and foundation for health research systems: Summary of the functions and operational components of health research systems.** Source: Modified from Pang et al. [7].

There is evidence [6] that to create an environment supportive of research requires some important national and institutional level factors necessary for the conduct and governance of health research, such as individual and institutional capacity strengthening, retention of skilled researchers, institutional collaboration and networks, among other factors. Such an environment has a beneficiary impact on building the evidence base to design health policies and improve the health of the population. There are many conceptual frameworks that describe the linkages between the different types of environments and how they impact research itself or the national health research system. For example, in McIntyre’s research capacity conceptual framework [6] (Figure 1), different environments interact with each other to influence the development of research capacity in a given country. An important catalyst of what is described as the task network is the Ministry of Health (MoH), which tends to influence the institutional, national and external research environments in the country. Pang et al. [7] also describe a framework (Figure 2) that supports the strengthening of national health research systems. The authors contend that having the elements of Pang’s and McIntyre’s frameworks available and properly functioning, creates an enabling R4H environment. They therefore sought to explore the existence of these within the government ministries often directly responsible for R4H in the Economic Community of the West Africa States (ECOWAS) region.

The MoHs are expected to play a key role in the governance, coordination, funding and creation of the demand for research to solve the health systems’ problems. At the national level, the MoH are expected to lead in the development of national policy, strategic development, and priority documents or demands for research [8,9]. The coordination of the activities of stakeholders involved in research for health and the development of ethical oversight for health research activities should be driven by the MoHs. However, some studies conducted in Africa have shown several weaknesses in carrying out these roles [10-12]. These studies noted that there was often limited information on the structure and organisation of the direct agencies responsible for carrying out these governance and coordination activities within the MoH, and whether the structures had the ability to fulfil those functions. The authors believe that such understanding, along with the capacity of the personnel within these MoH structures, could help to explain some of the root causes of the weaknesses within the national health research systems to support research. There was also an interest in determining if there was dedicated funding within the MoHs to carry out these identified activities.

Recent expert recommendations to support research capacity strengthening in low- and middle-income countries are ‘to build comprehensive, holistic and demand-driven models of national health research systems which genuinely engage policymakers, government officials, the media, health-care professionals, private companies and insurers, patient advocacy groups, community-based organizations, and the general public, as well as the full spectrum of other social, cultural, civil society and faith-based institutions’ [13]. It is therefore vitally important to strengthen the governmental partners when creating this kind of a model. Health research utilization to drive the policy-making agenda and national health programs design [13] are also important.

As an interested partner, the West African Health Organisation (WAHO), a specialised health agency of the ECOWAS Commission, has as part of its mission the mandate to promote R4H in the ECOWAS region. It seeks to facilitate R4H through improved governance and management, advocating for increased research funding, strengthening individual and institutional research skills, and promoting and disseminating research results as proposed in the conceptual framework by Pang et al. [7]. The MoHs through which WAHO primarily works at the country level to implement its mission are therefore its important strategic partners. Thus, it is important for WAHO to clearly understand the environment in which health research is conducted in such countries especially from the perspective of the MoHs before the commencement of any directed interventions in the countries. WAHO therefore conducted an exercise to understand the structures that constitute the research for health environment, especially in the MoHs, and the strengths and weakness of the research systems in the various countries.

This paper focuses on the information gathered regarding the national R4H environment and the different organisational arrangements available within each of the MoHs for the management and governance of research. The process also served for all the representatives present to collectively document and understand the research environment in the region and begin to collectively and individually design and support interventions that would help improve their country-level R4H environment and the region as a whole. This work should serve as a foundation for any future review.

## Methods

The data was collected from country representatives of the research units of MoHs in 14 out of the 15 ECOWAS countries (Benin, Burkina Faso, Cape Verde, Côte d’Ivoire, Gambia, Guinea, Guinea Bissau, Liberia, Mali, Niger, Nigeria, Senegal, Sierra Leone, and Togo). Ghana was the only country in the region that was not able to participate in the data collection. The questionnaire was designed with reference to key publications and other relevant literature on R4H [6,7,10,12]. It was translated into the three official ECOWAS languages (French, English and Portuguese) for ease of comprehension and pretested amongst a small sample of the international WAHO staff, who were chosen to reflect all the linguistic groups. Information was collected on the national-level R4H environment (governance and management of research activities in the MoH, existence of a national policy, strategic and priorities documents; health research ethics committee; research funds; coordination structures; monitoring and evaluation systems; networking and capacity building opportunities).

Questionnaires were self-administered by heads of the country research units or their senior deputies who provided the required information within the MoH during a regional workshop. Each participating country also made a country report presentation of its R4H situation in the MoH. Following this process, a face-to-face discussion was held with all the respondents where completed questions were reviewed for completeness and accuracy.

Details of the information requested on the different sub-sections of the R4H environment covered in the questionnaire and during the workshop discussions are described as follows:

*Research governance body:* Data on governance body focused on the type of structure responsible for R4H management, the availability of terms of reference, the organizational chart of the institution or organization, who the unit managers are, the personnel training, and departmental functions.

*Policy and strategic orientation documents:* The documents reviewed included, where available, national health policies, national health development plans, health research plans, and other relevant documents.

*Health ethics:* The data requested on health ethics related to the availability of a national ethics committee, the extent to which the national ethics committee was functioning for health research, and ethics training for committee members within the country.

*Resource mobilisation:* The existence of a budget line within the budget of the MoH for the operation of research structure, the funding of research projects, the existence of strategies to implement the Mexico and Algiers declarations to allocate 2% of the budget of the MoH and 5% of the budget of health projects or programmes to research or training, the availability of a resource mobilisation document, and the availability of technical and financial partners to support the research for health Unit.

*Coordination:* Data was collected on the existence of a coordinating structure for R4H and the strengths, weaknesses, opportunities, and threats to improving coordination.

*Monitoring and evaluation:* Details of any existing mechanism to collect and share research results, the existence of a structure that synthesizes results for policy-makers, a monitoring and evaluation mechanism, the existence of indicators and of a programme for the management of health research information.

*Capacity building:* The availability of training grants for researchers, in-country post-graduate short-term training programs on research, and training on health systems research and ethics.

*Networking:* Data was collected on the number of partners, the extent to which the R4H unit participates in other networks, and the participation of the personnel of the structure in scientific meeting.

Information from other major contributors to the R4H environment in the country, for example the research institutions, researchers, media, NGOs, other governmental ministries, and their interaction with the MoH with respect to R4H activities was not collected and thus not discussed in this review.

The information generated by the questionnaire was put through a process of validation during the regional workshop organized for this purpose from the 7^th^ to the 9^th^ of February, 2011. The 14 representatives at the meeting included directors of training, planning and research units, heads of health development centres, or research technical advisors of MoH who filled the questionnaires. During the workshop, two group discussions were organized. The first group discussion focused on the strengths, weaknesses, opportunities, and threats (SWOT) and the lessons learned from the R4H environment in each country. In the second session of discussions, future capacity building and staffing needs were evaluated for each country with a view to identifying the gaps and reviewing plans to provide support (results are not presented in this paper). All the discussions were recorded and subsequently transcribed by Nvivo by two sociologists (ST, BK). The ‘Organizational Diagnosis Questionnaire’ tool developed by Robert Preziosi to identify strengths and weaknesses in the functioning of an organization and/or its sub-parts, was also used [14]. In this paper, only the results of descriptive analysis are presented. All the original data are password protected and stored at the Head Office of WAHO in Bobo-Dioulasso, Burkina Faso.

## Results

### Structure responsible for research for health (R4H) within the Ministry of Health

Table 1 presents the information on the structure responsible for R4H within the MoHs of ECOWAS countries. Various forms of structures in charge of research exist within the MoHs. In seven countries, there was a Directorate*,* which is a relatively self-reliant body with decision-making ability that oversaw health research governance and management in the country. In four countries, a research Division exists within a larger Directorate. Three countries had smaller research service units within what constituted a larger Division.

**Table 1:** Description of structures in charge of research within the Ministries of Health (MoHs), ECOWAS member states, 2012

	**(Number of countries**
	**out of fourteen)**
The structure in charge of research within the MoH	
Direction	7
Division	4
Service	1
Unit	2
Existence of **terms of reference** for the structure in charge of research	13
Existence of an **organogram** for the structure in charge of research	11
**The head** of the structure in charge of research is	
Medical Doctor	7
Another Health Official	7
The head of the structure in charge of research at the MoH is **experienced in the conduct of research**	12
Have staff of the department received **training** in the area of governance and management of health research	1
The **functions** of the structure in charge of research at the Ministry of Health are:	
a) To provide governance (**development of policy documents**) of the national health research system	14
b) To manage the national health research system (**planning and implementation of daily activities**)	13
c) To **coordinate research activities**	**14**
d) To ensure **capacity building** of the actors in the system	11
e) To ensure **monitoring and evaluation** of the system activities	11
f) **Conduct research** for the MoH	9
Existence of a **budget line for the structure in the budget of the MoH**	**5**
Existence of **technical and financial partners** working with you in the research department	6
Is the research department **taking part in a multi-country project?**	**7**
Does the research department **belong to a network?**	**5**
Does the network **promote capacity building?**	**6**
Do the heads of the research departments **attend national/regional/international conferences on health research?**	**13**

In 13 countries, the structure had defined terms of reference. All the countries, with the exception of one, also had clearly defined organizational charts providing clarity for the units reporting and business relationships within the MoH. All the countries had some form of national health policy document (standalone health policy or health write-up as a part of a broader national vision document). Half of all the research governance structures were headed by a medical doctor and in the other half (seven countries) by a scientist or health-related professional, e.g., biologist and health service administrator. In 12 countries, the officials in charge of these units had some personal experience pertaining to the conduct of research. None of the heads of these R4H units in the MoH, except one, had benefited from some form of training in health research management. In all countries, the profile, competences and qualifications required for the head of the structure responsible for research within the MoH was not defined. During the follow-up group discussion, the participants suggested the following profile to be used as the minimum qualifications required for such a position:

“A Bachelors degree plus five years postgraduate experience as a minimum; be trained in the area of research ‘but not necessarily a senior researcher’; be trained in administration, management or health structures management, information and communication technologies, advocacy, leadership, group facilitation, quality outcomes; be able to work under pressure, collaborate with policy-makers and administrative officers and finally have a holistic view of health.”

The current functions assigned to the research units in the MoH include development of policy documents and coordination of health research activities in all the countries, management of research in 13 of the 14 countries, ensuring capacity building, monitoring and evaluation in 11 of the 14 countries, and conducting research on behalf of the MoH in 9 of the 14 countries. Only five countries had a budget line for directly financing the activities of the research unit in charge of these R4H activities. In six countries, this structure also had a partner for technical and financial support.

Indeed, of the 14 structures in charge of R4H represented, 7 had participated in a multi-country project and 6 had taken part in networking activities on a routine basis with the aim of fostering capacity building. In 13 out of 14 countries, the heads of these structures had opportunities to attend symposia on R4H at the national, regional and/or international level.

A further assessment of the state of the R4H infrastructure and the necessary personnel required to carry out its activities was made during group discussions. Thirteen of the 14 countries present had high-speed Internet connectivity available for their use. In all the 14 countries, all the personnel had functional computers. The number of staff in the structure of research at the MoH level varied from 1 in Mali to 8 in Nigeria.

Table 2 shows information on the existence of political and strategic documents, and R4H ethical, financial and coordination issues under the MoH. Monitoring, evaluation and capacity building issues are also covered in this table.

**Table 2:** Guidance and management documents for research activities within the Ministries of Health of the ECOWAS member states, 2012

	**(No of countries**
	**out of fourteen)**
**Existence of political and strategic documents**	
Existence of a **national health policy**	**12**
Existence of **a strategic plan for health development**	**12**
**Is research taken into account** in the policy documents and strategic plans	13
Existence of any **policy and strategic documents for research development**	8
Existence of any **document on research priorities**	**7**
**Ethics Committee on Research for Health**	
Existence of a **National Ethics Committee on Research**	**12**
Is the **National Ethics Committee on health research functional**	**12**
Were **members** of the National Ethics Committee on health research **trained**	**8**
Existence of any ethics **training framework** (workshops, courses, etc.) in the country	7
**Mobilization of funds**	
Existence of a **budget line for the financing of research**	**5**
Existence of any **document on resource mobilization for research**	**0**
Is there a national approach to the implementation of the recommendations of the commitment made by the Health Ministers to allocate 2% of the national health budget and 5% of health projects and programs to research	5
**Coordination**	
Existence of a research coordinating structure within the MoH	**8**
Existence of a consultation framework for stakeholders in health research	6
**Monitoring and Evaluation**	
Existence of **mechanism to synthesize and share research results** in the country	6
Existence of a **structure that summarizes research findings for policy making**	**4**
Existence of **monitoring and evaluation mechanisms for health research**	**3**
Existence of **indicators for the monitoring and evaluation** in use for health research in the country	3
Existence of program **on information management for health research** in your country	2
Existence of any **monitoring and evaluation reports**	2
**Capacity Building**	
Existence of **training grants** for researchers	2
Existence of **short-term/postgraduate courses** in the country for researchers	3
Existence of any **operational research course** for the staff in the MoH	5
Existence of any **course on ethics** in the country	7

### Existence of national R4H policy and strategic documents

A national health policy and a strategic health development plan existed in 12 (85.7%) of the 14 ECOWAS countries present, and R4H was broadly taken into account in these documents in all these countries. Only eight countries had specific R4H policy and strategic documents, while seven countries had specific documents on health research priorities. These country priorities did not differ significantly from one another and are therefore probably a good summary of the research priorities of the ECOWAS region. They included health determinants, communicable and non-communicable diseases, health systems challenges, traditional medicine, management and quality of services, health economics, reproductive health, and child mortality and morbidity. However, participants noted that little consideration was given to these research priorities by externally funded studies carried out in their countries.

### Functional national ethics committees

Twelve countries had national ethics committees that held regular sessions to oversee study protocols. However, only five of the countries had members of their National Ethics Committees trained in research ethics. Half of the countries had some framework for training members of the committees on the activities and management of health research ethics, which was either by means of workshops or courses.

### Funding of research for health

Amongst the ECOWAS countries, funding of R4H has been inadequate and difficult to mobilize despite the intent of the countries to implement the Algiers and Bamako Declarations. In 9 out of the 14 countries present, there was no budget line in the MoH’s budget for R4H activities and where this existed the funds allocated were still very small compared to external funding. In Côte d’Ivoire for instance, public funding for R4H represented less than 1% of the country’s health budget. In 2008, in Burkina Faso, foreign partners funded 87% of research for health projects.

All the countries lacked a coherent resource mobilisation strategy or policy document. Five countries had taken steps to implement the recommendations of the commitment of Ministers of Health to allocate 2% of the national health budget and 5% of the budget of health projects and programmes to research.

### Coordination, monitoring and evaluation, and health research information dissemination mechanisms

Health research governance and management requires the coordination, monitoring and evaluation of research activities, as well the dissemination of research findings. While there was at least a coordinating structure for the R4H activities in the MoHs in eight of the countries present, only six had a stakeholder consultation framework and a mechanism for synthesizing and sharing research findings in the country. Six out of the fourteen countries had a structure or mechanism to synthesize research findings and, of these, only four specifically summarized research findings for policy-makers. Only three of the countries had monitoring and evaluation mechanisms or health research target indicators. Two countries indicated they had some reports on the monitoring and evaluation of research activities. Only two countries also had programmes on information management for health research.

### Capacity building for researchers

To ensure that researchers are able to carry out quality studies, it is recommended that they build their capacities and improve the technical facilities of their research institutions. It was observed, through the analysis of the opportunities available to do this at the MoH level, that only two of the ECOWAS countries had grants for training of researchers, three countries had short-term postgraduate training programs, and five had operational research training courses. Half of the countries had some course on ethics (Table 2). Other weaknesses also reported during group discussions included inadequate training of young researchers in scientific and research protocol writing, poor intra- and inter-institutional collaboration among research teams, and the lack of clearly defined career paths for researchers working at the MoH.

## Discussion

This paper highlights some strengths and weaknesses in the R4H environment under the MOH’s control in the ECOWAS region. The strengths include the existence of a formal structure for governance and management of R4H activities with clear terms of reference, and the existence of ethical review committees in most of the ECOWAS countries. The weaknesses generally included the lack of R4H strategic documents, funding support for the R4H structures, and general support for capacity building for both researchers and the R4H units. There was minimal coordination in the research activities to address or reflect their national priorities, or the promotion of the use of research results to influence policy decisions. Several reasons have been suggested for the minimal uptake of research for policy making in low- and middle-income countries, and this has called for effective communication to meeting the need of the targeted audience with research results [9,15]; the capacity to do so must be continually built in the R4H management units of the MoHs.

### Institutional capability of the R4H units within the MoHs

Only 50% of the countries had a directorate in charge of R4H within the MoH. This proportion is less than what was reported by Kennedy et al. [12] in Mediterranean countries, where 7 out of 10 countries had a directorate in charge of R4H. The difference between the two regions could be due to the level of research for health development between the Mediterranean region and West African countries.

To give adequate emphasis to the role of research within MoHs, the R4H units should ideally be at the level of a Directorate. This would facilitate the proper functioning of the R4H unit and influence decision making and policy drafting across all the different Directorates or Ministries to use research. Operating at the level of a Directorate would afford the R4H unit an opportunity to have a dedicated budget line for its operations and also make it a little easier to track that country’s contributions toward R4H. This directorate also tends to have cross cutting roles in the activities of other directorates thus fostering more collaboration within the ministry. However, no information was available to suggest that having a Directorate would necessarily ensure efficient research management in the country. All the countries have the sovereign right to decide on the structures of their institutions, however, when countries give prominence to research and have an equivalent of an R4H directorate in the MoH, this provides enormous benefits for all the other health activities in the country compared to countries that do not. There were also other indirect downstream benefits affecting the health policies and practices implemented across the health activities in the country and thus ultimately benefiting the peoples of the country. These were the consensus views of the participants during the discussions.

Continued capacity building for R4H unit staff is important for their proper functioning. Some of the required competencies can be obtained through health research management training, which could be done at the ECOWAS regional level because of the similarity of many of the national structures and their national research priorities. This would also enable networking, experience and information sharing among the different countries because of their shared aspirations, further allowing peer countries to plan, monitor and evaluate the progress of the various health research activities and to benchmark their activities to countries in the region with similar goals and experiences.

HRWeb, a research management platform developed by the Council on Health Research for Development (COHRED: http://www.cohred.org) and tested in Senegal, can be used to facilitate R4H information sharing among the countries and their partners. The different in-country partners involved in research could help put in place this system to easily allow the sharing of administrative documents, policy documents, structures and the status of on-going research projects, as well as research results. Policymakers across the region could use the information generated from the regional peer countries and around the world to inform and improve their decision-making and policy development. This platform was presented to the participants during the regional workshop.

### National research for health strategic documents

As compared to previous studies conducted in Africa [10,12], more countries (87%) in the ECOWAS region now have policy documents and strategic development plans for R4H, showing that progress has been made in this region. Currently, there is a regional project funded by the International Research Development Centre (IDRC) and WAHO whose aim is to help countries lacking these documents to develop them. This project is currently being implemented by WAHO with technical support from COHRED and was started in 2011, and is expected to run until 2014. One country had already developed and adopted its research for health policy and development plan a year after this project’s implementation. In addition, at the end of this workshop, all countries that did not have the various strategic documents and were not part of the above regional project expressed the desire to develop them.

Regarding research priorities, it was not clear how or when these priorities were derived and if they were a reflection of the disease burden or priority health sector problems. It was also not obvious how researchers and their funders knew about their existence, thereby explaining why it did not reflect in the proportions of the research outputs.

### Funding of research for health

Funding for research is crucial as it allows for the strengthening of researcher and institutional capacities to conduct research. The funding source, however, plays a key role in determining the content of research as health researchers often conduct activities based in some part on the donor’s agenda. One of the most commonly reported weaknesses of R4H activities in Africa is low national funding [10,12,16,17]. This observation was affirmed in the results as very few countries had budget lines to support the activities of the R4H structures in the MoH to fund research projects, especially those of national interest. Only a third of the countries had mechanisms in place to help implement the international recommendations approved by Ministers of Health during the Mexico Summit and reaffirmed in Algiers. These recommendations called for the allocation of 2% of the budgets of MoHs and 5% of the budget for health projects/programmes to research at the 58^th^ session of the World Health Assembly [18]. None of the 14 countries in attendance had a specific strategy document on how to mobilize resources for research.

### Health research ethics

The findings showed that almost all the ECOWAS countries had some form of research ethics committee that was operational. Kirigia et al., in a study conducted in the member states of the WHO African Region, found that out of 28 respondent countries, 64% (18/28) confirmed the existence of a health ethics committee [11]. The finding in the ECOWAS region is therefore encouraging and shows the progress that has been made by the countries in this area. However, additional efforts ought to be made to train the members of Ethics Committees. There are currently several training opportunities available in the countries, and online; ethics committee members should be encouraged to avail themselves of the various training opportunities. Regional training opportunities and support should be shared widely to further improve the R4H environment. Specific minimum and continuous training requirements should be prescribed for members of the ethics committees because of the constantly changing context of the health research environment as well as different aspects of research ethics management.

### Research coordination, monitoring/evaluation and information dissemination mechanism

As Kennedy et al. [12] showed in their study of Mediterranean countries, monitoring and evaluation of research activities is also currently inadequately carried out amongst ECOWAS countries. This state of affairs also applies to synthesizing research findings and the dissemination of activities; these different elements should be included in building the institutional structures within MoHs. In this regard, Hyder et al. [19], while recognizing the complexity of the interface between policy-making and research in low-income countries, proposed the involvement of four key actors in the promotion of the use of health research (the government, health providers, scientists, and the community). The commitment of governments or decision-makers is extremely important. In this regard, the international community frequently organizes meetings for decision-makers such as the Mexico Ministerial Summit on health research in 2004 [20]. There are also the scientific experts who, with the policy-makers, should maintain continuous communication amongst themselves to help foster the R4H environment. Health service providers and the media also play important roles in the development and implementation of health policies. Finally, the involvement of the community, who are the ultimate beneficiaries of the health system, is vitally important in fostering this interface in research dissemination and utilization.

Ssengooba et al., in Uganda, took a different approach and explained that the factors facilitating the implementation of the policy on the prevention of mother to child transmission of HIV (PMTCT) for example, and the continuous use of research to improve upon programmes were to be grouped under the following categories: ‘Common platforms for learning and decision-making’, ‘implementation of pilot project aiming at assessing the feasibility of the intervention’, ‘collaboration with specialized institutions in research to conduct operational research’, and ‘visibility of benefits’ [21]. It is therefore important for the countries to clearly identify their paradigm of operation in how to facilitate the use of research results to drive health policy.

### Capacity building

The financial support allocated for building the capacities of researchers and the other research related professionals in the MoHs is low. This may be related to poor overall local funding for research within the countries. To address this inadequate funding issue, north–south, south-south and north–south-south partnership strategies have been put in place in several resource-limited countries. For example, in South Africa, Airhihenbuwa et al. described the importance of United States-South African partnership in training thirty postgraduate students in two South-African universities by building their capacities to analyse HIV-related stigma in the national context [22]. Also in the area of HIV and AIDS control, the south-south partnership between Brazil, Cuba, Mexico and some African countries led to training programs for health professionals and technicians in various domains: production of generic ARV, and the development strategies for prevention and care of HIV [23]. In Zimbabwe, support was provided from the Danish schistosomiasis laboratory to strengthen the capacities of the Zimbabwean Blair Research Laboratory through the provision of doctoral level training by the biomedical research-training institute, a sub-regional institute [24]. To alleviate this weakness, WAHO has, since 2009, also provided some scholarships for graduate level training in health research. There are also other types of south-south and north–south cooperation amongst the different ECOWAS countries but these are insufficient to meet all the needs required to improve the R4H environment. The European Union’s seventh framework research programme funding has also benefited some of the countries in the region (e.g., Ghana through the COHRED-led MASCOT project). Another often neglected area of health research capacity building is in traditional medicine and herbal pharmacopeia, which is currently being supported by WAHO [25,26].

### Partnership and networking

Partnership and networking ability is immensely important for a R4H management unit. The WHO, during its 63^rd^ World Health Assembly advised member states to increase inter-country cooperation to achieve efficiency in the area of health through the sharing of experiences, data and information on best practices and resources, the pooling of training resources, and the use of common and standardized evaluation methods. From the data received, this recommendation had been partly implemented in seven ECOWAS countries.

### Opportunities

This process has allowed the authors to explore various opportunities within and between the countries to further improve the research capacity in the region. It has also spurred many collaborative projects that are currently under review. Overall, this baseline has been very useful in doing the SWOT analysis of the MoH R4H environment to design targeted interventions.

### Strengths and limitations

This process had the advantage of combining several techniques including formal country report presentations of their R4H environment, and the use of self-administered questionnaires and group discussions to triangulate the information. The ability for the information to be immediately validated by a feedback mechanism during the meeting helped provide a comprehensive picture of the R4H environment and activities. Although the questionnaires were self-administered, the team was available to assist in providing the necessary clarifications on information that may have been lost in translation and to receive immediate feedback on responses. This ensured a 100% response and completion rates on the questionnaires from all the respondents, which contrasts with two similar surveys using self-administered questionnaires sent via diplomatic mails to 46 WHO African Region’s member states that recorded a response rate of 21.7% (10/46) [10] and 60.9% (28/46) [11]. Finally, the results of the questionnaires were collectively reviewed by the participants themselves thus providing an immediate feedback mechanism illustrating the strengths and gaps in each country’s environment. This also provided an environment for the R4H managers to explore areas of cooperation amongst the countries.

The process had some limitations. The self-administered questionnaires and group work focused mainly on the profile of the managers of the structure in charge of health research, though this structure also includes other staff whose competences are also needed for its operation. This workshop only targeted the structure’s managers or their senior representatives who had to provide answers pertaining to several aspects of the R4H governance and management. Research governance and management certainly involves many other actors including academia, civil society representatives and members of national health ethics committees who may provide different perspectives on the different issues under consideration. This was also a cross-sectional analysis of the R4H activities in the region and does not reflect or explain the historical or the socioeconomic context of the current structures. It also does not give a plan or explain the rate and direction of evolution of the R4H environment in the different ECOWAS countries. Some countries were clearly ahead of others in the different aspects and evolution of the research for health activities. There was heavy reliance on the information provided by the participants as the heads or officers in charge of research in the MoH, and it was not immediately possible to independently verify responses provided in the countries themselves.

This analysis also does not examine the entire research infrastructure beyond the MoHs and how they influence the components of the R4H environment. The majority of R4H activities (research projects, funding, publication, etc.) likely occur outside the MoH. Linguistic and geo-political considerations, as they affect the R4H environment, were not examined in this review.

## Conclusions

The focus of this paper was to provide a description of the state of the national R4H environment in the MoHs within the ECOWAS region. This assessment has shown that the R4H enabling environment within the MoHs of ECOWAS member states is not perfect; however, there is a desire for improvement and some of the basic building structures already exist. Strengthening capacities and funding opportunities are required to create a future with an enabling environment for the conduct and use of health research. This would require some harmonisation activities, capacity building, management structures and oversight, and networking opportunities. Harmonized structures, working in partnership or in network may contribute to improving the situation in the future. WAHO, with its political mandate and its programme for facilitating research in the ECOWAS countries, and in partnership with the various actors in the research field, could facilitate the harmonization of underlying structures, and advocate for greater importance and significant funding for research within MoHs. Moreover, by organizing regional meetings, WAHO could facilitate the training of the various personnel in the various aspects of R4H as well as facilitate experience sharing among countries within the sub-region. Some of these partnerships are already in place and should be strengthened to improve research for a better health status of the West African people.

## Abbreviations

BRL: Blair Research Laboratory; COHRED: Council on Health Research for Development; ECOWAS: Economic Community of West African States; HIV: Human Immunodeficiency Virus; IDRC: International Development Research Centre; MASCOT: Multilateral Association for Studying Health Inequalities and enhancing North–south and South-South Cooperation; MoH: Ministry of Health; PMTCT: Prevention of mother to child transmission of HIV; R4H: Research for Health; SWOT: Strengths, Weaknesses, Opportunities and Threats; WAHO: West African Health Organisation; WHO: World Health Organization.

## Competing interests

IS, JA and SSK work with the West African Health Organisation and have since 2011 been working on the IDRC sponsored West African Research for Health project. The opinions expressed in this paper are solely that of the authors and not official position of their institution. The other authors declare that they have no competing interests.

## Authors’ contributions

SI made substantial contribution to the conception of the study design organized the writing of the manuscript. AJ, KB and SDT assisted in the analysis of the data, writing and review of the article. AJ and SI revised several versions of the manuscript. KSS provided support and the supervision of the study design and review of paper. All authors read and approved the final manuscript.
